# Environmental Determinants of Infectious Disease: A Framework for Tracking Causal Links and Guiding Public Health Research

**DOI:** 10.1289/ehp.9806

**Published:** 2007-05-31

**Authors:** Joseph N.S. Eisenberg, Manish A. Desai, Karen Levy, Sarah J. Bates, Song Liang, Kyra Naumoff, James C. Scott

**Affiliations:** 1 Department of Epidemiology, University of Michigan, Ann Arbor, Michigan, USA; 2 School of Public Health, University of California Berkeley, Berkeley, California, USA; 3 Department of Environmental Science, Policy, and Management, University of California Berkeley, Berkeley, California, USA; 4 Division of Environmental Health Science, The Ohio State University, Columbus, Ohio, USA

**Keywords:** environmental change, infectious disease, interdisciplinary, system theory, transmission dynamics

## Abstract

**Background:**

Discoveries that emerging and re-emerging pathogens have their origin in environmental change has created an urgent need to understand how these environmental changes impact disease burden. In this article we present a framework that provides a context from which to examine the relationship between environmental changes and disease transmission and a structure from which to unite disparate pieces of information from a variety of disciplines.

**Methods:**

The framework integrates three interrelated characteristics of environment–disease relationships: *a*) Environmental change manifests in a complex web of ecologic and social factors that may ultimately impact disease; these factors are represented as those more distally related and those more proximally related to disease. *b*) Transmission dynamics of infectious pathogens mediate the effects that environmental changes have on disease. *c*) Disease burden is the outcome of the interplay between environmental change and the transmission cycle of a pathogen.

**Results:**

To put this framework into operation, we present a matrix formulation as a means to define important elements of this system and to summarize what is known and unknown about the these elements and their relationships. The framework explicitly expresses the problem at a systems level that goes beyond the traditional risk factor analysis used in public health, and the matrix provides a means to explicitly express the coupling of different system components.

**Conclusion:**

This coupling of environmental and disease transmission processes provides a much-needed construct for furthering our understanding of both specific and general relationships between environmental change and infectious disease.

Public health scientists are increasingly discovering that the recent emergence or re-emergence of infectious diseases has an origin in environmental change (McMichael and Martens 2002; Morse 1995; Patz et al. 2000). These environmental changes encompass social processes such as urbanization and creation of transportation infrastructure, as well as ecologic processes such as land and water use, bio-diversity loss, and climate change. Concern surrounding these trends has inspired much exploratory research because these phenomena are often anthropogenic, interrelated, and accelerating. Yet there remains a pressing need to more clearly define the causal relationships, leading from a distal environmental change to alterations in more proximal environmental characteristics and disease transmission cycles, which eventually lead to a shift in the prevalence, distribution, or severity of an infectious disease (Figure 1).

In this article we focus on the intermediary relationships between proximal environmental characteristics and transmission cycles. Environmental sciences have traditionally focused on the links between distal environmental changes and their effects on proximal environmental characteristics, whereas public health scholarship has focused on the link between transmission cycles and disease burden. We argue and provide a framework for leveraging the wealth of prior research in both realms by highlighting the links between them. These links are conveniently defined through a matrix formulation in which system elements from one component are mapped onto system elements from another component. The matrix cells can then be used to provide information on what is known about the particular link. This matrix formulation is consistent with a dynamic systems approach that accounts for feedbacks, a central feature of complex systems.

The Environmental Change and Infectious Disease (EnvID) framework uses a systems theory structure to integrate and analyze disparate information from a variety of disciplines. Our ultimate goal is to identify knowledge gaps and define research directions as well as to develop relevant study designs and approaches for data analysis so that knowledge about environmental change can be incorporated appropriately into the study and control of infectious diseases. In the ensuing section, we survey the literature on contemporary frameworks of environmental change and infectious disease. Next, we motivate and describe the EnvID framework. We then use this framework to generate a putative matrix of plausible relationships between proximal environmental characteristics and transmission cycles. This matrix can be used to assess the strengths and weaknesses of existing knowledge and thus prioritize avenues for future research.

## Contemporary Frameworks of Environmental Change and Infectious Disease

During the modern era of public health, attention to the natural and built environment has fluctuated, reflecting wider trends in biomedical thought and praxis (McMichael 2001; Porter 1999). In the 19th century, the progenitors of public health instituted a suite of interventions that astutely reflected perceived linkages between environmental conditions and poor health. Campaigns that focused on sanitation, hygiene, housing, and nutrition led to unparalleled leaps in health and longevity (Szreter 1988). Despite a flawed rationale based on theories of miasma or contagion, these campaigns effectively controlled many significant communicable pathogens (Cipolla 1992). Moreover, their success demonstrated the utility of intervening further up the causal chain, even in the absence of comprehensive knowledge (Smith and Desai 2002).

Subsequent advances in germ theory gradually overshadowed the environment as a major cause of disease. In the 20th century, public health strategies for the control of infectious disease progressed along a reductionist trajectory that emphasized vaccines, antibiotics, pesticides, and barriers to infection. These technologies resulted in further improvements in the public’s health and deservedly continue to influence much of biomedicine.

However, a growing body of literature on environmental change and infectious disease has emerged during the past decade, returning public health to its roots (Daily and Ehrlich 1996; Epstein 1995; Gratz 1999). Overviews on the topic have permeated a growing array of academic fields (Anderson 2004; Cohen 2000; Kombe and Darrow 2001; Price-Smith 1999) and popular literature (Garrett 1994). These commentaries have raised interest and stimulated research, but understanding how environmental change impacts an infectious disease process remains a challenge. This challenge hinders efforts to translate research into public health policy and practice.

To help bridge this gap, we highlight three threads of scholarship that link environmental change and infectious disease:

- debates on the future of epidemiology,
- integrative reviews on environmental change and infectious disease, and
- mathematical models of disease transmission.

We draw and build on the major themes and converging concerns and approaches within these threads.

### Debates on the future of epidemiology

Suggestions that public health move from a discipline concerned primarily with risk factors at the individual level toward one concerned with multiple levels and types of causation have prompted vigorous discussions. Several themes within these debates on the future of epidemiology offer guidance for the study of environmental change and infectious disease.

#### Strengths and weaknesses of risk factor–based analysis

Risk factor analysis has become virtually synonymous with modern epidemiology. It supplies the theoretical and methodologic foundation for studying relationships at an individual level, and within this realm, provides the basis for testing causal hypotheses. Although risk factor analysis has enjoyed much success, its limitations have come to light in recent years (Pearce 1996; Susser 1998). In response, more valid and precise techniques that better account for bias and error have been developed (Greenland 2001; Lash and Fink 2003; Robins et al. 2000). Others, on the other hand, have continued to advocate the risk factor approach, stressing the role of apparently inexplicable results in eventually guiding discovery (Greenland et al. 2004; Savitz 1994).

Although such refinement and reflection have addressed some weaknesses of risk factor analysis, others have emerged. For example, although the individual level may be an important scale for probing certain public health questions, risk factor analysis is challenged by the complexity of fundamental causes, including social and ecologic drivers (Krieger and Zierler 1996; Pimentel et al. 1998), gene–environment interactions (Hunter 2005), and life-course trajectories (Susser and Terry 2003). Risk factor analysis, even with modification, faces limits in its capacity to examine causal mechanisms at multiple scales (Susser and Susser 1996a); it may adeptly explain who is at risk but not why risks exist or differ within and between populations (Krieger 1994; Rose 1985; Susser 2004).

#### Causal inference for infectious disease

Yet other critiques have questioned the traditional analytical approach in epidemiology that assumes independence of outcomes. The assumption of independence means that the causal link between exposure and disease is made at the individual level. This model hinges on the conjecture that populations are simple collections of individuals, and the nature or arrangement of interactions between individuals does not alter patterns of risk (Koopman and Lynch 1999). The propagation of exposures and outcomes through a population, however, is intrinsic to most communicable pathogens and plainly violates the so-called stability assumption, which requires independence among individuals’ exposure and outcome status (Halloran and Struchiner 1995). Disease (e.g., cholera) influences exposure (e.g., contaminated water source), which in turn influences outcome (e.g., more cholera), and so on, via transmission. It is not simply an individual’s exposure to water that alone determines the individual’s outcome but rather the exposure and outcome status of all other individuals in possible prior contact with the same water source (Eisenberg et al. 1996).

**Figure 1:**
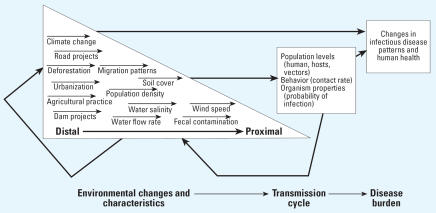
Environmental determinants of infectious disease (EnvID) framework.

Feedbacks among exposures and outcomes generate context-dependent effects. Population-level effects are not equivalent to the sum of individual-level effects, and individual-level effects depend on the distribution of population-level effects. Herd immunity and threshold density are two well-known examples of this phenomenon. Moreover, feedbacks are also integral to many wider causal webs of environment and disease (Figure 1). In complex systems, inappropriate inferences based on potential outcomes can severely distort the interpretation of effects and misdirect the application of interventions (Eisenberg et al. 2003; Halloran and Struchiner 1995; Jacquez et al. 1994). Risk factor analysis for infectious disease can sometimes be partially salvaged through conditioning on transmission potential (Haber 1999) or employing counterfactuals (Robins et al. 2000), but results from both experimental and observational studies warrant cautious scrutiny prior to generalization.

#### New paradigms for epidemiologic research

The impetus to understand causality within complex systems has inspired the search for new paradigms that do not abandon conventional research but rather situate it within the study of processes. Several more sophisticated approaches have been proposed, the most influential of which include ecoepidemiology (Susser and Susser 1996b), social-ecologic systems perspectives (McMichael 1999), and eco-social theory (Krieger 2001). These efforts all use a systems theory–based approach to extend the purview of causation across axes of space, time, and organizational level and propose to interrelate research at different scales through feedbacks and interactions.

### Integrative reviews on environmental change and infectious disease

In recent years, research on the linkages between environmental change and infectious disease has proliferated, embracing multiple types and levels of anthropogenic disturbance, pathogenic process, and scientific approach. Integrative reviews on environmental change and infectious disease have played a critical role for the nascent field by distilling results from disparate sources. Because of space constraints, we cannot systematically assess these integrative reviews, and our reference list overlooks many worthwhile publications. Instead, we concentrate on three emerging trends within this literature of notable import to future projects and syntheses.

#### Conceptual frameworks

A set of integrative reviews articulate conceptual frameworks for comprehensively organizing knowledge about systems of interacting components that link fundamental drivers to disease resurgence through an interplay of subsystems (e.g., social, economic, biological, physical) (Barrett et al. 1998; Cohen 1998; Daszak et al. 2000; Mayer 2000; Weiss and McMichael 2004; Wilcox and Colwell 2005). Some existing conceptual frameworks could also be applied to environmental change and infectious disease. Particularly germane are frameworks for climate change (Colwell 1996; McMichael and Butler 2004; Patz et al. 1996), globalization (Woodward et al. 2001), social epidemiology (Diez-Roux 2000; Subramanian 2004), and environmental health (Black 2000; Parkes et al. 2003). The various conceptual frameworks reveal the exceptional complexity and difficulties of their subject matter, such as striking a balance between the general versus the specific, and difficulty in assessing validity and relevance to decision-making bodies. Still, conceptual frameworks undoubtedly encourage critical thought and shape the evolution of the field.

#### Interdisciplinary research and integration

Virtually all integrative reviews are, at least to some extent, interdisciplinary, as the study of environmental change and infectious disease clearly requires expertise from numerous fields. Most integrative reviews include various biomedical sciences but selectively emphasize certain social or ecologic sciences, with more recent work displaying greater inclusivity and deeper collaboration. In addition, integrative reviews that reference the gradually growing number of case studies on sustainable development (Corvalan et al. 1999; Shahi et al. 1997) or ecosystem approaches (Corvalan et al. 2005; Parkes et al. 2005; Patz et al. 2004) bridge scientists, policymakers, activists, and citizens.

#### Categorization schemes

Explicitly or implicitly, many integrative reviews deploy particular typologies to categorize environmental changes and/or infectious diseases. Most schemes do not emphasize the most salient features of environment–disease relationships. Infectious diseases are commonly grouped according to scientific taxonomy or clinical symptoms, which might be useful for purposes of diagnosis and treatment but do not correspond reliably to environmental drivers. Wilson (2001) groups infectious diseases by transmission cycle, an approach we adopt here. As environmental change involves complex causes and consequences, proposed typologies also have been elusive. Still, the tentative discrimination of environmental changes along continuums of spatial extent, temporal persistence, distal to proximal action, and social versus ecologic impact could more usefully translate linkages, as they are identified, to a putative causal network.

### Mathematical models of disease transmission

Mathematical models of disease transmission began to be developed nearly a century ago with work on mass action (Ross 1915) and threshold densities (Kermack and McKendrick 1933), with subsequent elaboration from mathematical and population biologists (Anderson and May 1991). Ecologists, epidemiologists, and mathematicians are increasingly deploying transmission models toward informing study designs, effect estimates, and intervention strategies (Eisenberg et al. 2002; Levin et al. 1999). From the extensive literature on transmission models, we underscore two important and related conclusions: *a*) transmission models are instructive as a well-developed systems theory–based approach, and *b*) transmission models can themselves be incorporated into wider studies of environmental change.

#### Systems theory–based approach

The overt consideration of feedbacks and interactions within and between populations in a transmission model allows for a consideration of infectious diseases as inherently dynamic and interdependent processes, and thus causality as context dependent and systems based (Koopman 2004). Transmission models elucidate the relationships governing the creation and distribution of risks by disentangling individual- and population-level effects (Halloran et al. 1991). The insights enabled by this analysis are often nuanced. For example, altering the pattern of connections between exposed and unexposed individuals may impact the level of infection within a population more so than altering the exposure status of individuals in that population (Koopman and Longini 1994). If a core group is sustaining infection in a larger group, targeting interventions based on individual-level risk factors will not, in general, address the principle cause of disease (Christley et al. 2005; Jacquez et al. 1988; Verdasca et al. 2005).

#### Transmission models embedded within wider systems

The influence of social and ecologic contexts on disease transmission has been recognized for diseases spread through direct contact [e.g., sexually transmitted diseases (STDs) and airborne diseases] (Klovdahl et al. 2001; Rothenberg et al. 1998; Shen et al. 2004), diseases with environmental reservoirs (e.g., waterborne diseases) (Colwell 2004; Eisenberg et al. 2005), and diseases for which land use change modulates vector populations (e.g., vectorborne diseases) (Lindblade et al. 2000; Ostfeld and Keesing 2000). Transmission models can serve as conceptual or analytical instruments to analyze the interactions between environmental contexts and transmission cycle components (McMichael 1997; Smith et al. 2005).

### Preliminary synthesis

These three threads of scholarship all advocate a gradual shift toward a systems theory–based approach. The emerging epidemiologic paradigms—spurred by debates on the future of epidemiology—and the conceptual frameworks—distilled from integrative reviews on environmental change and infectious disease—are essentially extensions of the systems perspective. This systems perspective is intrinsic to mathematical models of disease transmission that spans the gulf from distal environmental change to disease burden by bringing together strengths from interdisciplinary fields and sound causal inference.

We propose a series of steps, derived from these three threads, toward constructing a more robust framework. An initial step defines flexible and logical classifications of environment and disease that can readily translate to causal webs. These classifications or components form the basis of our framework. A second step begins to integrate transmission with environment by examining the intersection of proximal environmental characteristics and transmission cycles and acknowledging the useful but limited insights of risk factor analysis. Here we detail some of the connections that exist between environment and health. A third step develops causal networks with explicit feedbacks and interactions that highlight the dynamic properties of this large-scale environmental process.

## A Framework to Contextualize the Environmental Determinants of Infectious Disease

**Figure 2:**
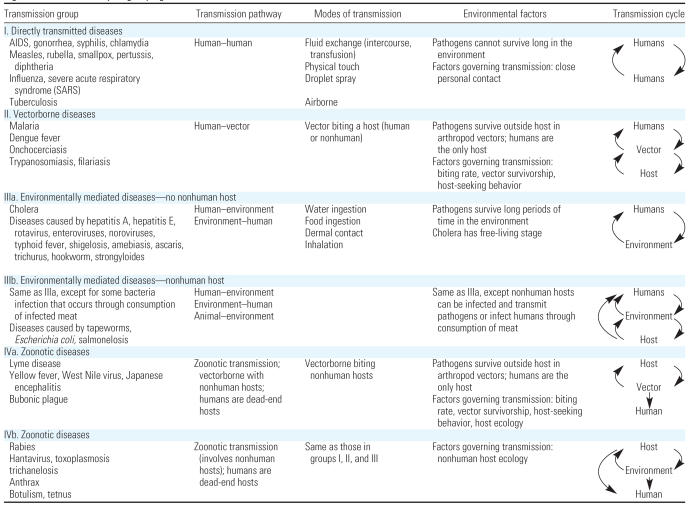
Transmission cycle groupings.

The EnvID framework encompasses three interlocking components: environment, transmission, and disease (Figure 1). There has been a tendency to delineate environmental changes into those that are social, such as urbanization, and those that are ecologic, such as deforestation, but in actuality any process affecting human health has both social and ecologic components that are inextricably linked. These changing environmental processes may affect the transmission cycles of infectious pathogens. We present six transmission groups that each relate to the environment in distinct ways (Figure 2). Disease burden is determined by incidence and severity of infection, which is in part a function of the transmission cycle.

As an initial step to put this framework into operation, we propose using a matrix formulation to move both backward toward the more fundamental causes of disease, and forward toward disease burden. A matrix, as described in this section, can provide an explicit description of the interconnections between system elements. In this manner the matrix defines one component of the system and provides a means to summarize what is known and what is unknown about that component.

This section describes each of the three main EnvID components, with a focus on the linkage between proximal environmental characteristics and transmission cycles.

### Framework description

#### Environmental change

Although the environment represents the first component of the systems-level EnvID framework, it is itself a system of interacting components. We choose to disaggregate the environment into distal environmental changes that act on disease transmission through multiple intermediate steps and proximal environmental characteristics that directly affect disease transmission.

The list of distal environmental changes in Table 1 includes anthropogenic changes that affect landscape ecology, human ecology, and human-created environments as well as natural perturbations and natural disasters. There are clear interactions among these distal factors and their effects. For example, climate change may impact the characteristics of El Niño, roads may contribute to urbanization, deforestation may amplify climate change, and the impacts of natural disasters might be augmented by anthropogenic changes such as loss of wetlands.

**Figure 3:**
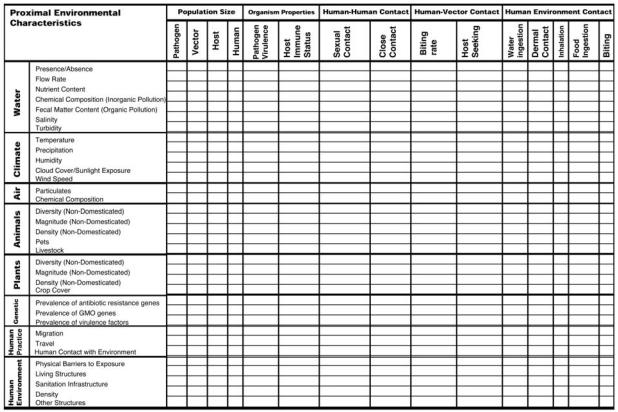
Matrix for mapping the relationship between proximal environmental characteristics and transmission cycles. GMO, genetically modified organism.

The distal changes are larger in temporal and spatial scales than the more proximal environmental characteristics that they influence. Proximal environmental characteristics are defined as directly measurable physical, chemical, biological, or social components of the environment, including populations and traits of relevant organisms (Figure 3, column 1). Proximal environmental characteristics can have a direct influence on the environment of the organisms in question (pathogen, vector, host, or human) and thus may directly affect the transmission cycle of an infectious disease.

Distal changes affect disease only through a series of causal linkages. For example, a dam does not change health directly; rather, a dam causes changes in water flow, which may affect mosquito habitat, which in turn can affect transmission potential of malaria. A new road may affect disease through major demographic shifts that ultimately lead to increased sexual activity and STD incidence. The causal linkages between distal and proximal, therefore, represent a continuum, and the labeling of a factor as distal or proximal is relative. However, by focusing on measurable proximal environmental characteristics studies can more clearly and definitively describe the causal linkages that changes in the environment have on disease transmission.

#### Transmission cycles

The impact of proximal environmental characteristics on disease burden is mediated through transmission cycle dynamics. We categorize pathogens into one of six transmission system groups based on their distinct relationships with the environment (Figure 2).

The first group (I) includes person-to-person transmitted diseases, wherein “contact” between humans is the principle mode of transmission, through intimate proximity (e.g. casual contact or droplet spray) or bodily fluid exchange (Mandell et al. 2000). In this group, humans are the only host and the environment does not serve as a reservoir for the pathogen. The second group (II) includes all vectorborne diseases in which humans play an important role in the transmission cycle. Transmission occurs through contact between humans and vectors (defined here as arthropods that move pathogens from one host to another). The third group includes infectious diseases for which the environment (e.g., food, water, soil) plays a significant role in a pathogen’s transmission cycle. In the first subtype (IIIa), transmission occurs between humans and the environment directly; no other host animals are involved. In the second subtype (IIIb), nonhuman hosts mediate transmission, although the environment remains an integral part of the transmission chain. The fourth group (IV) includes all pathogens that cause zoonotic diseases. The transmission cycles of all zoonotic diseases share two key features; humans are dead-end hosts and no person-to-person transmission is possible. Subtype IVa includes vectorborne zoonotic diseases. Nonvectorborne zoonotic diseases in which pathogens are transmitted indirectly through the environment or directly from a host are included in subtype IVb.

While each of these six transmission cycles describes a different mechanism of transmission, they share common attributes; namely, all are affected by the population level and/or density of the host and/or vector, and all are driven by a transmission potential governed by a number of biological and environmental characteristics. The transmission rate from one host to another can be thought of as the product of two processes: contact rate and infectivity. The contact rate quantifies the interaction between hosts or between a host and the environment and is generally determined by host behavior and properties of the environment. Infectivity, or probability of infection given contact, is a function of both the virulence of the pathogen and the immune status of the host. Environmental changes can affect population levels of the host, vector, or environmental stage of the pathogen as well as the transmission rate at which pathogens move between hosts, vectors, and environment.

#### Disease patterns and disease burden

Understanding how environmental change affects disease transmission and incidence does not address the crucial public health concern of disease burden. For example, high levels of rotavirus disease exist in both developed and developing countries, but the mortality rates in developing countries are much higher than in developed countries (Parashar et al. 2003). In addition, environmental change can affect disease burden directly without necessarily influencing transmission. If environmental change affects nutrition, for example, this can in turn affect disease severity. Disease burden can also feed back to transmission cycles, as people who are more seriously sick may have higher pathogen loads.

### Relating proximal characteristics to transmission cycles

Many studies have focused on the association between specific proximal changes in the environment and health, and how these proximal characteristics influence transmission. Because proximal environmental changes often affect transmission processes directly, experiments can be designed to elucidate these mechanistic relationships. These proximal environmental characteristic/transmission cycle (PEC/TC) relationships can be mapped using a tabular “transmission matrix,” in which the environmental proximal characteristics are represented as rows and the transmission cycle characteristics are represented as columns (Figure 3).

The transmission matrix organization is consistent with two paradigms prevalent in the literature. First, the classic paradigm of infectious disease transmission depicts the agent, host, and environment as each representing one node of a triangle. The matrix columns represent the host and agent nodes. They consist of population/demographic factors such as density, virulence, and immune status, as well as those factors that influence the rate of transmission from one host to another, such as ingestion rate, vector biting rate, and human-to-human contact rates. The matrix rows represent the environment node that consists of those specific proximal environmental characteristics that can affect host/agent properties.

The proximal environmental characteristics, represented as rows in Figure 3, were chosen to encompass physiochemical characteristics associated with air, water, and climate; ecologic characteristics of plants and animals; genetic characteristics of pathogens; and human characteristics associated with short- and long-term human migratory patterns, human contact with the environment, and social structure. Again, each row may not be relevant for every infectious disease, and the list is not meant to be definitive. The choice of columns is based on the transmission system paradigm elaborated above. This paradigm suggests that disease incidence is proportional to the population level of all organisms that can harbor the pathogen, and the transmission rate, which is the product of the rate of contact between hosts or between a host and the environment, and the probability of infection given a contact occurs. The matrix columns therefore represent factors needed to estimate the transmission rate, and the matrix rows represent those environmental factors that can impact the transmission potential by modifying factors represented in the columns. Each cell represents the potential for a proximal environmental characteristic to affect a component of the transmission cycle. Different portions of the matrix (columns and rows) will apply to the different transmission groups outlined in Figure 2.

Environmental change will obviously impact disease patterns differently depending on the transmission cycle of a particular pathogen (Figure 2). Because diseases in transmission group I are directly transmitted between humans, they are most influenced by the proximal changes in the environment that affect human social structure, such as conditions of severe overcrowding, social changes affected by access to transportation, and migration and travel patterns. However, many of these pathogens can survive in the environment for hours or more, and therefore other physiochemical characteristics of the environment may also play a role. Environmental change can impact transmission of diseases in transmission group II through its effects on proximal factors associated with vector ecology, such as vector biting behavior, mortality, and population density, or through social changes that can increase human contact with vectors. Because all pathogens in transmission group III can survive in the environment and some have nonhuman hosts, environmental change impacts transmission through modifying human exposure to contaminated media such as drinking water, recreational water, and food, animal hosts, and other infectious individuals. Because this class of pathogens consists of both vectorborne and environmentally mediated pathogens, the contact patterns of transmission group IV are similar to those of groups II and III, but transmission is sustained in nonhuman hosts, so environmental factors associated with the ecology of these nonhuman hosts and their relationships to pathogens are most salient.

**Figure 4:**
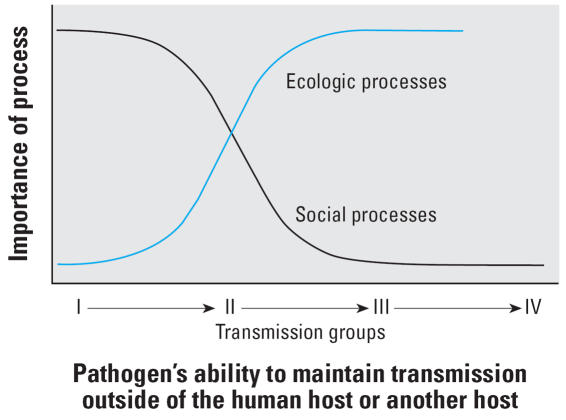
Importance of ecologic and social processes from different transmission groups.

These differences in the role of social and ecologic processes in mediating environmental change between the six transmission groups are represented in Figure 4. Environmental change impacts those diseases caused by pathogens within transmission group I via mechanisms that are primarily mediated by social processes. In contrast, those changes impact diseases caused by group IV pathogens via mechanisms primarily mediated by nonhuman ecologic processes. Both ecologic and social processes influence groups II and III pathogens.

#### Application of framework: road development and diarrheal disease transmission

The EnvID framework can be used in several ways. For example, it can be used to assess all possible impacts of environmental factors on a single infectious disease. A formal use of this framework to conduct a systematic review, evaluating the weight of evidence of how the environment affects a representative pathogen for each of our transmission groups, is forthcoming in a future article. The framework can also be used to guide a particular research question exploring the impacts of a distal environmental change on a particular disease. It provides a structured way to conceptualize the causal network, which can guide research approaches.

To illustrate this latter approach, we present a short case study here that examines the proposed causal linkages between road development and diarrheal disease. In 1996 the Ecuadorian government began a 100-km road construction project to link the southern Colombian border with the Ecuadorian coast. The road was completed in 2001, but secondary roads continue to be built, linking additional villages to the paved road. These roads provide a faster and cheaper mode of transportation compared with rivers and have led to major changes in the ecology and social structure of the region (Sierra 1999). Although there is evidence that road construction affects the incidence of vectorborne and sexually transmitted diseases (Birley 1995), the impact that environmental changes from road construction have on diarrheal disease remains largely unexplored. A PEC/TC matrix of this environmental change/infectious disease example illustrates that there is strong risk factor evidence for the relationship between the proximal factors of water quality as well as sanitation and hygiene levels and transmission of enteric pathogens. There are fewer studies that demonstrate a relationship between distal social factors such as crowding or general social infrastructure and distal ecologic factors such as regional-scale water patterns with diarrheal disease (Curriero et al. 2001).

**Figure 5:**
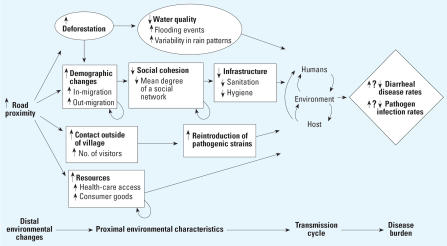
Causal diagram of the relationship between roads and diarrheal disease.

Road development represents a comparatively distal environmental change that can impact both ecologic processes, such as deforestation, biodiversity, and hydroecology, as well as social processes, such as migration, demographics, and infrastructure. Deforestation can cause major changes in watershed characteristics and potential local climate change, which can affect the transmission of enteric pathogens (Curriero et al. 2001). Perhaps more important than ecologic processes, social processes such as migration that are facilitated by roads can increase the rate of pathogen introduction into a region. Road proximity affects short-term travel patterns, thereby resulting in continual reintroduction of new pathogen strains into communities. New communities are created along roads, and existing communities can rapidly increase in density. These changes in social structures of communities often create or are accompanied by inadequate infrastructure, which affects hygiene and sanitation levels, and in turn the likelihood of transmission of enteric pathogens. Roads can also increase flows of consumer goods such as processed food, material goods, and medicines and may also provide communities with increased access to health care, health facilities, and health information. Figure 5 illustrates a mapping of the distal environmental change, due to road proximity, to the proximal environmental factors associated with water sanitation and hygiene that directly influence disease transmission. As pointed out in the figure, whether these relationships result in an increase or decrease in disease burden is not known.

The framework and matrix help elucidate the necessary interdisciplinary research elements and approaches needed to study environmental impacts of road development on diarrheal disease transmission in this Ecuadorian landscape. The research question requires a design that examines and integrates processes at multiple spatial and temporal scales using regional, village-wide, individual-, and molecular-level data, and system-level models to integrate these data. Epidemiologic study designs are complemented by hydrology and water quality studies, remote sensing and geographic information system technologies, social network analysis, ethnography, and molecular-strain typing to elucidate pathogen flow across the landscape. The scale and inherent dimensionality of the problem requires this systems-based approach to examine relationships between environmental, social, and biological change to explain the detection of the relationship between road access and infection.

## Conclusion

As public health moves more toward examining how both ecologic and social processes affect disease transmission, and more specifically toward examining the fundamental role of environmental change in creating the landscape of human disease, a systems theory framework is needed from which to integrate and analyze data obtained from the disparate but relevant fields of study involved. As the review of contemporary frameworks suggests, the inherent multidimensionality of these problems precludes the use of standard analytic approaches.

The EnvID framework builds on previous frameworks by *a*) articulating a broad but flexible and logical system specification; *b*) explicitly incorporating transmission groups that provide important links to public health intervention strategies; *c*) emphasizing the intersection of PECs and these transmission processes; *d*) incorporating a matrix formulation that specifies system components, identifies knowledge gaps in the literature, and facilitates the integration of an existing body of research; and *e*) emphasizing dynamic processes and hypothesis generation. The EnvID framework attempts to facilitate the integration of a body of research, and in so doing, identifies the source of disputes and prioritizes avenues for resolution. As research advances, the EnvID framework can help integrate the various factors at play in determining environment–disease relationships and the connections between them.

A systems-based approach serves to explicitly emphasize the reality that studies are embedded within a wider web of interactions, especially studies relevant to environmental change and infectious disease. This systems-based approach can be put into operation by the proposed matrix formulation. The matrix formulation represents a succinct way to characterize the system, providing information on the interrelatedness of the different system components and defining research needs. Data needs for the matrix often will be a combination of site-specific data, collected specifically for the systems-based analysis, and data from the literature, which always need to be assessed with respect to quality and appropriateness. The matrix can additionally be used to conduct model-based simulation studies that may provide *a*) valuable information on the broader system’s dynamics associated with specific, more focused, empiric studies; and *b*) a means to integrate and contextualize these empiric studies with other processes that are either more distal or proximal to disease burden.

A formal use of this framework to conduct a systematic review, evaluating weight of evidence of how the environment affects a representative pathogen for each of our transmission groups, is forthcoming in a future paper. The challenge for future studies on the environmental determinants of disease is to develop new approaches for thinking about processes at the system level that in turn will elicit new study designs and data analysis. Given the increasingly explicit nature of the connections between proximal environmental change and health—for example, the SARS epidemic in 2003, the Indian Ocean tsunami in 2004, Hurricane Katrina in 2005, and the recent focus on avian influenza—now is the time to synthesize these connections in order to move this important field of environment and health forward.

## Figures and Tables

**Table 1:** Examples of distal environmental changes and diseases they may impact.

Environmental change	Description	Disease
Hospitalization	Increased people and time spent in hospitals	Tuberculosis (TB) Enteric and respiratory diseases
Urbanization	Increasing migration to and growth within towns	Dengue fever Diseases caused by fecal–oral pathogens Diseases caused by TB
Antibiotic usage	Emergence of antibiotic-resistant strains of bacterial pathogens	Multidrug resistant TB and salmonelosis *Salmonella typhimurium*
Water projects	Water flow changes due to dam construction and irrigation networks	Schistosomiasis Malaria
Agricultural intensification	Changing crop and animal management practices; fertilizer and biocide use; use of genetically modified organisms	Cryptosporiosis Diseases caused by *E. coli*
	Increased interplay between humans and domesticated animals	Influenza, severe acute respiratory syndrome (SARS), avian flu
Deforestation	Loss of forest cover, changing water flow patterns Reforestation and human encroachment along and into forested areas	Malaria Lyme disease Hemorrhagic fever AIDS
Transportation projects	Construction of roads, increasing access to remote areas	Malaria STDs
Natural perturbations	Large-scale climate and other changes such as El Niño events	Cholera and leptospirosis
Cataclysmic events	Localized landscape changes caused by earthquakes, tsunamis, large fires, and other	Water-related diseases like cholera
Climate change	Changing temperature and precipitation	Malaria, dengue fever, and schistosomiasis
